# Immunogenicity in Mice Immunized with Recombinant Adenoviruses Expressing Varicella-Zoster Virus Envelope Glycoprotein E

**DOI:** 10.3390/v15122288

**Published:** 2023-11-22

**Authors:** Yanpeng Zheng, Lei Huang, Huiru Ding, Huawei Xu, Rigan Shu, Jiemei Yu, Xianglei Peng, Yuanhui Fu, Jinsheng He

**Affiliations:** College of Life Sciences and Bioengineering, Beijing Jiaotong University, Beijing 100044, Chinajmyu1@bjtu.edu.cn (J.Y.);

**Keywords:** herpes zoster, recombinant adenoviruses, immune pathways, immunogenicity

## Abstract

Herpes zoster (HZ) is a disease caused by the reactivation of latent varicella-zoster virus (VZV). The subunit vaccine, Shingrix^®^, and live attenuated vaccine, Zostavax^®^, could be used as an HZ vaccine that prevents HZ from being developed due to the reactivation of latent VZV in the sensory ganglia due to aging, stress or immunosuppression. In this study, the recombinant adenoviruses rChAd63/gE expressing glycoprotein E (gE) of VZV based on chimpanzee adenovirus serotype 63 (ChAd63) were constructed and investigated for the immunogenicity of different immune pathways in C57BL/6 mice. The results showed similar CD4^+^ T and CD8^+^ T cell responses to Shingrix^®^ were induced in mice vaccinated using rChAd63/gE via different immune pathways. This study elucidates that recombinant adenoviruses expressing VZV gE could be appropriate for further development as a new HZ vaccine candidate via different immune pathways.

## 1. Introduction

Varicella-zoster virus (VZV) is a lymphotropic and neurotropic human herpes virus that causes chickenpox (varicella) after initial infection and remains latent in the sensory and autonomic ganglia for life after recovery. Following the reactivation of latent VZV in later life due to aging, stress or immunosuppression, herpes zoster (HZ) will develop [1]. Glycoprotein E (gE) is a conserved protein between genotypes and the most abundant glycoprotein in the envelope of VZV, which is crucial for virus replication and cell–cell transmission [2,3]. The gE protein can induce gE-specific cell-mediated immunity (CMI) and humoral immunity. In particular, the gE-specific and CD4^+^ CMI plays an important role in preventing the symptoms of HZ [4,5]. Therefore, gE has become the main target antigen of the HZ vaccine.

So far, two licensed vaccines are available to prevent HZ: a live attenuated vaccine, Zostavax^®^, and a recombinant subunit vaccine, Shingrix^®^ [6,7]. Studies have shown that both vaccines induced cellular and humoral immunity and significantly reduced the incidences of HZ and postherpetic neuralgia (PHN) in the elderly. Zostavax^®^ reduced the frequencies of HZ by 51.3% and PHN by 66.5%, but its preventive effect on HZ declined with age. The published reports have already described the deaths of six people caused by viral sepsis with the live attenuated vaccine, Zostavax^®^, who lived in countries with excellent healthcare systems: Canada, England, Sweden and Australia [8,9]. So, sale and use of the live attenuated vaccine, Zostavax^®^, were discontinued in the United States on 1 July 2020 [7,10,11,12,13]. In sharp contrast, the overall protective effect of Shingrix^®^, composed of VZV gE protein and the AS01B adjuvant, in people aged over 50 years is 97.2% and does not decrease with age [14,15,16]. However, approximately 10% of the vaccinees had a grade 3 systemic reaction (unable to perform usual daily activity), caused by the ASO1B adjuvant, not the gE protein in the vaccine, that lasted 1 to 2 days. Therefore, it is still necessary to produce a herpes zoster vaccine with fewer adverse effects at the time of injection.

The primary infection of VZV can induce VZV-specific immunity, and a high level of VZV-specific antibodies can persist in the host for life [17]. Further studies showed that VZV-CMI is the essential component in the recovery of VZV infection, the restriction of latent VZV reactivation and the prevention of HZ [18,19,20,21,22]. Moreover, the level of VZV-CMI is also closely related to the severity and even the occurrence of HZ [5,22,23,24,25,26,27]. VZV-CMI mainly involves CD4^+^ and CD8^+^ effector and memory T cells, as well as natural killer cells. Natural killer (NK) cells restrict VZV replication by secreting antiviral molecules (IFN-γ and granulysin) or via direct cytolysis [28,29]. However, VZV-specific CD4^+^ T cells producing IFN-γ function both as helpers and for major histocompatibility class II (MHCII)-restricted cytotoxicity, suggesting that the VZV-specific CD4^+^ T cell response should be the preferred endpoint for the development of an HZ vaccine [30,31].

Adenovirus-based vectors are considered effective vaccines due to their powerful ability to deliver genes into host cells, strong adjuvant activity, high production, safety profiles and ease of storage [32]. Importantly, adenovirus vectors express antigenic proteins de novo in infected cells, which induces Th1-biased immune responses and strong CMI responses to adenovirus-based vaccines [33]. Examples of evoking such immune response are documented against respiratory syncytial virus (hRSV) and severe acute respiratory syndrome coronavirus 2 (SARS-CoV-2) [34,35]. Principally, an adenovirus-vectored HZ vaccine candidate is very plausible to induce the anticipated and critical CMI preventing the reactivation of the lurking VZV and the resulting HZ. Therefore, in this study, recombinant adenoviruses rChAd63/gE expressing the VZV gE protein were constructed and prepared. In order to avoid the inhibitory effect of the pre-existing high-titer neutralizing antibody against adenovirus on the immunogenicity, chimpanzee adenovirus serotype 63 (ChAd63) with low seroprevalence were selected here. The SARS-CoV-2 vaccines ChAdOx1 nCoV-19 (AstraZeneca) and Ad26.COV2.S (Janssen) have been associated with rare cases of vaccine-induced immune thrombotic thrombocytopenia (VITT) [36]. Therefore, the safety of the adenovirus vector vaccine has been widely considered. The occurrence of VITT was related to the infiltration or infusion of adenovirus into the vein caused by intramuscular injection, and the activation and damage of platelets and endothelial cells, or the formation of complexes with platelet factor 4 (PF4) leading to immune damage, etc. [36]. Therefore, VITT can be completely alleviated or avoided via the intranasal inoculation of Ad-vectored vaccines [37]. Intradermal (i.d) injection can trigger systemic immunity [38], and has the longest absorption time among all parenteral routes because there are fewer blood vessels and no muscle tissue. The efficacy of this new microneedle-based immunization approach is due to the presence of several types of immune cells (such as dendritic cells (DCs), T lymphocytes, NK cells, macrophages and mast cells) in the epithelium. In order to avoid VITT and broaden the immune pathway of the adenovirus vector vaccine, we investigated whether C57BL/6 mice immunized intranasally (i.n) or i.d with rChAd63/gE can achieve similar immune responses as with intramuscular (i.m) injection. So, the immunological effects in C57BL/6 mice immunized intranasally (i.n), intramuscularly (i.m) or i.d with rChAd63/gE were investigated using homologous prime-boost vaccination strategies. The results showed i.n, i.m or i.d immunization with rChAd63/gE induced similar humoral and cellular immune responses to Shingrix^®^ in mice.

## 2. Materials and Methods

### 2.1. Cells and Viruses

Human embryonic kidney cells (HEK293) were maintained in Dulbecco’s Modified Eagle Medium (DMEM, HyClone Laboratories, Logan, UT, USA) supplemented with 10% fetal bovine serum (FBS, Life Technologies Australia Pty Ltd., Thornton, NSW, Australia), 100 IU/mL penicillin and 100 μg/mL streptomycin (1% P/S, Mediatech Inc., Manassas, VA, USA), as well as 2 mM L-glutamine (Amresco, Solon, OH, USA), in a humidified incubator at 37 °C, 5% CO_2_.

**Figure 1:**
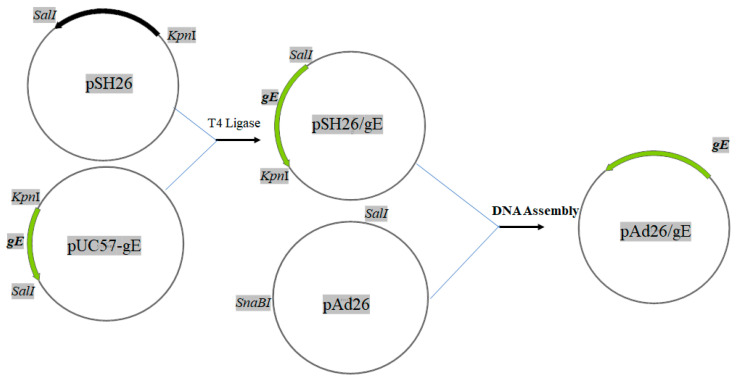
Construction of recombinant adenovirus plasmid pChAd63/gE.

The recombinant adenoviruses rChAd63/gE, expressing gE of VZV based on ChAd63, were constructed and rescued as described previously. The gE open reading frame (ORF) (GenBank: DQ452050.1) was cloned into shuttle plasmid pShuttle63. Then, the expressing cassette was cloned further into pChAd63 to produce pChAd63/gE (Figure 1). The pChAd63/gE was linearized via digestion using restriction enzyme PacI and transfected into 293 packaging cells to generate rChAd63/gE. The rChAd63/gE was purified by CsCl ultracentrifugation. The titers of the purified rChAd63/gE were measured using the Qubit fluorometric quantitation (Thermo Fisher Scientific, Waltham, MA, USA) method and Adeno-X rapid titer (Clontech, Mountain View, CA, USA) method. To purify the rChAd63/gE, the HEK293 were infected with rChAd63/gE at 10 MOI and incubated for 60–72 h at 37 °C. Then, the cells were collected and centrifugated at 4000 rpm at 4 °C for 10 min, and resuspended in 10 mL 100 mM Tris-HCl (pH 8.0). After the cells were lysed with 1 mL 5% sodium deoxycholate, DNAase I was added to the cell lysate, and the supernatants were collected at 3000× *g* for 15 min at 4 °C and layered on top of a discontinuous CsCl gradient composed of 1.35 g/mL CsCl and 1.25 g/mL CsCl in a P40ST rotor (Hitachi, Tokyo, Japan). The gradients were ultracentrifuged at 35,000 rpm for 1 h at 4 °C. The fluffy layer at the interface between 1.25 g/mL to 1.35 g/mL CsCl was collected and transferred into an ultracentrifuge tube, and the gradients were ultracentrifuged at 35,000 rpm, 4 °C for 16–20 h in the P55ST rotor (Hitachi). The fluffy layer was collected and dialyzed with Slide-A-Lyzer (Thermo Fisher Scientific, Waltham, MA, USA) in 10 mM Tris-HCl for 24 h at 4 °C. After purification, the titers of the purified rChAd63/gE were measured using Qubit^®^ dsDNA HS Assay Kits (Thermo Fisher Scientific) and the Adeno-X rapid titer (Clontech, Mountain View, CA, USA) method, and the expression of gE was detected using Dot blotting and indirect immunofluorescence.

### 2.2. Qubit Fluorometric Quantitation

The purified rChAd63/gE was appropriately diluted (e.g., 20-fold dilution) with the virus lysis buffer (0.2% SDS TE solution (10 mM Tris-HCl, 1 mM EDTA, pH 8.0) with a final concentration of 0.1 mg/mL Protease K). After incubation for 2 h at 56 °C, the sample was briefly shaken and centrifuged instantaneously, and 190 μL dsDNA buffer containing 0.5% fluorescent dye was added into a 10 μL sample. After oscillating and mixing, the mixture was placed at room temperature for 2 min. The sample concentration was measured using a Qubit^®^ 3.0 Fluorometer.

### 2.3. Adeno-X^TM^Rapid Titer Procedure

A total of 2.5 × 10^5^ HEK-293 cells were plated in 24-well plates. After 4 h, serially diluted rChAd63/gE was added to 24-well plates with HEK-293 cells. After incubation for 48 h at 37 °C, the medium was aspirated, and the cells fixed using ice-cold 100% methanol for 20 min at −20 °C. Then, the plates were blocked with 1% (*w*/*v*) BSA in PBS at 37 °C for 2 h. An anti-ChAd63 antibody dilution was added to the wells and incubated for 1 h at 37 °C. The plates were washed again, and HRP-conjugated IgG antibodies were added (Santa Cruz Biotechnology, Santa Cruz, CA, USA) and allowed to incubate for 1 h. Finally, the plates were washed and developed with 100 μL 3,3,5,5-tetramethylbenzidine (TMB; Sigma, St. Louis, MO, USA) substrate solution. A minimum of three fields of positive cells using a microscope with a 20× objective was counted, and the mean number of positive cells in each well was calculated. The infectious units (IFU)/mL for each well were calculated as follows:

$$\mathrm{IFU}/\mathrm{mL}=\left(\frac{(infected\;cells/field)\;\times \;(fields/well)\;}{volume\;virus\;(\mathrm{mL})\;\times \;(dilution\;factor)}\right)$$

### 2.4. In Vitro gE Protein Expression

The HEK-293 cells were infected with rChAd63/gE. At 48 h after infection, the cells were collected, washed twice with cold PBS and lysed with RIPA and analyzed using Western blotting. Protein separation was performed under reducing conditions (with 100 mmol/L 2-mercaptoethanol and boiling) on sodiumdodecyl sulfate 10% polyacrylamide gels. After blotting onto nitrocellulose membranes, the proteins were incubated with a mouse-specific antibody against gE (ab272686, Abcam, Cambridge, UK) and goat anti-mouse IgG-HRP (Dako, Hamburg, GER), and visualized using a two-color infrared fluorescence imaging system (Odyssey CLx, LI-COR Bioscience, NE, USA). The lysates from the uninfected HEK-293 cells and HEK-293 cells infected with rAd26/gE were used as negative and positive loading controls, respectively.

A total of 2.5 × 105 HEK-293 cells were plated in 24-well plates. After 24 h, 1 moi rChAd63/gE was added to the 24-well plates with the HEK-293 cells. After incubation for 24 h at 37 °C, the medium was aspirated, and the cells processed using ice-cold methanol containing 0.1% triton. The plates were blocked with 1% (*w*/*v*) BSA in PBS at 37 °C for 2 h. The anti-gE antibody dilution was added to the wells and incubated for 1 h at 37 °C. The plates were washed again, and cy3-conjugated IgG antibodies were added and allowed to incubate for 30 min. After washing, DAPI working solution was added and incubated at room temperature for 8 min in the dark. The images were observed using a fluorescence microscope.

### 2.5. Animal and Immunization

The SPF female C57BL/6 mice, aged 6–8 weeks, were purchased from Vital River Laboratory Animal Technology Ltd. (Beijing, China), maintained under SPF conditions and housed in Beijing Laboratory Animal Research Center. All experimental procedures were reviewed and approved by the Animal Welfare and Research Ethics Committee of Beijing Laboratory Animal Research Center (BLARC-JSB-DW/013-jl/001). Forty BALB/c mice were used for immunization experiments and divided into four groups of rChAd63/gE (i.n), rChAd63/gE (i.m), rChAd63/gE (i.d) and Shingrix^®^. The mice were immunized with rChAd63/gE (1 × 10^8^ PFU/mouse) in 50 µL or i.m with Shingrix^®^ (5 μg/mouse) in 50 µL on weeks 0 and 3 [39].

### 2.6. Enzyme-Linked Immunosorbent Assay (ELISA) for gE-Specific Antibodies

Blood was obtained from the retro-orbital plexus using a capillary tube, and collected in an Eppendorf tube. After centrifugation (5000× *g* for 15 min), the sera was stored at −20 °C. Then, 40 ng purified gE (Abcam) was adsorbed onto ELISA plates (NEST, Wuxi, China) overnight in PBS at 4 °C and blocked with 10% FBS in PBS at 37 °C for 2 h. Then, the serially diluted sera were added to the plates and incubated for 1 h at 37 °C. After the plates were thoroughly washed using PBST, HRP-conjugated anti-mouse IgG was further added into the plates (1:5000, Biodragon Immunotechnologies, Beijing, China), and allowed to incubate for another 1 h at 37 °C. Finally, 100 μL of TMB soluble (Biodragon Immunotechnologies) was used for the color reaction, and the reaction was terminated via the addition of 50 μL 2 M H_2_SO_4_. The absorbance was measured at 450 nm using a BioTek plate reader (BioTek, Winooski, VT, USA).

### 2.7. Flow Cytometry

Spleens from the vaccinated mice were harvested at 2 weeks after booster immunization and placed in mouse lymphocyte separation medium. The spleens were triturated and ground gently using cell strainers (Becton-Dickinson, San Jose, CA, USA) to obtain single-cell suspensions. The single-cell suspensions were centrifuged at 800× *g* for 30 min. Then, the splenocytes were collected and washed using complete RPMI 1640 medium (Invitrogen, Carlsbad, CA, USA). A total of 1 × 10^6^ splenocytes were plated in 96-well U bottom plates and centrifuged at 3000 rpm for 2 min, and stimulated with 10 μg/mL pooled peptides and brefeldin A (Biolegend, San Diego, CA, USA) overnight at 37 °C with 5% CO_2_. The cells were then stained using FITC anti-mouse CD4 antibodies (Biolegend) and PerCP/Cyanine5.5 anti-mouse CD8a antibodies (Biolegend), followed by intracellular staining using APC anti-mouse IL-2 antibodies (Biolegend) and PE anti-mouse IFN-γ antibodies (Biolegend). The samples were run on a BD FACSCalibur 2# (Becton, Dickinson and Company, Franklin Lakes, NJ, USA) and analyzed using the FlowJo software v10 (BD Bioscience, San Jose, CA, USA).

### 2.8. Statistical Analysis

Statistical analysis was performed using the SPSS software version 21 (SPSS, Chicago, IL, USA), and the means of multiple groups were compared using one-way analysis of variance with Tukey’s multiple-comparison test. *p* < 0.05 was considered significant.

## 3. Results

### 3.1. In Vitro Characterization of rChAd63/gE

**Figure 2:**
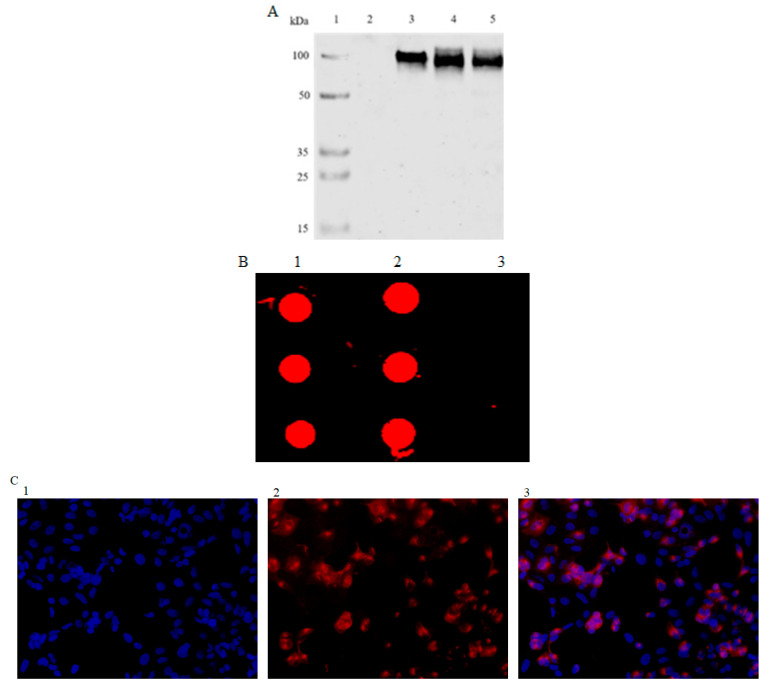
Analysis of the expressed gE protein from the recombinant adenoviruses. (**A**) The expression of gE was detected using Western blotting. 1: Marker (M), 2: Uninfected HEK293 cells (Negative control, NC), 3: HEK293 cells infected with rAd26/gE, 4: HEK293 cells infected with rChAd63/gE, 5: Positive control, Shingrix^®^. (**B**) The expression of gE was detected using Dot blotting. 1: Positive control, Shingrix^®^, 2: HEK293 cells infected with rChAd63/Ge, 3: Uninfected HEK293 cells (Negative control, NC). (**C**) The expression of gE was detected using indirect immunofluorescence. 1: DAPI staining, 2: Indirect immunofluorescence using anti-gE antibody and cy3-conjugated IgG antibody, 3: Result of merging 1 and 2.

To make clear the characteristics of rChAd63/gE in vitro, the HEK293 cells were infected with rChAd63/gE, the cells were collected, lysed and analyzed for the expression of gE protein using Western blotting under reducing conditions and the expressed gE protein could be observed as anticipated. The lysates from the uninfected HEK293 cells and Shingrix^®^ were used as negative and positive controls, respectively (Figure 2A). HEK293 cells were infected with rChAd63/gE, the cells were collected and analyzed for the expression of gE protein using Dot blotting and the expressed gE protein could be observed as anticipated (Figure 2B).

### 3.2. gE-Specific IgG Responses Induced by Immunization with Recombinant Adenoviruses

**Figure 3:**
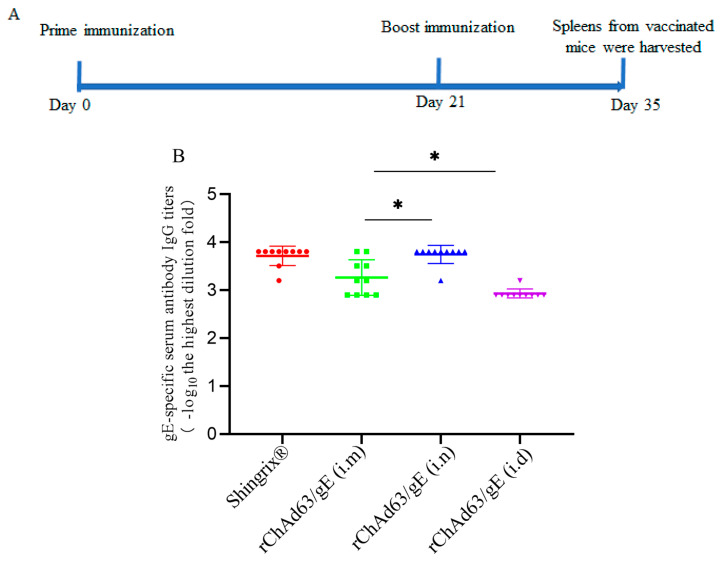
gE-specific IgG in C57BL/6 mice induced by the recombinant adenoviruses. (**A**) The experimental scheme. (**B**) gE-specific IgG in C57BL/6 mice induced by the recombinant adenoviruses. C57BL/6 mice (n = 10) were immunized with recombinant adenoviruses using homologous prime-boost scheme, with animals being boosted at 3-week intervals following the first immunization. On the 14th day after first immunization and homologous booster immunization, sera were collected, and gE-specific IgG was detected using ELISA. Data were one of two independent experiments and shown as mean ± SD, *: *p* < 0.05.

To determine the ability of rChAd63/gE to induce a specific antibody response against gE, we compared the gE-specific IgG responses following a homologous prime-boost strategy with rChAd63/gE (Figure 3A). The C57BL/6 mice were immunized with i.n, i.m or i.d with 50 µL rChAd63/gE (1 × 10^8^ PFU/mouse) or 50 µL Shingrix^®^ (5 μg /mouse), respectively, and antibodies against gE were measured using ELISA (Figure 3B). Compared with the i.m injection group, a higher level of gE-specific IgG was induced in the i.n immunization group (*p* < 0.05), while a lower level of gE-specific IgG was induced in the i.d injection group (*p* < 0.05).

### 3.3. Efficient Induction of gE-Specific IFN-γ and/or IL-2-Secreting CD4^+^ T Cells in Mice Immunized with Recombinant Adenoviruses

**Figure 4:**
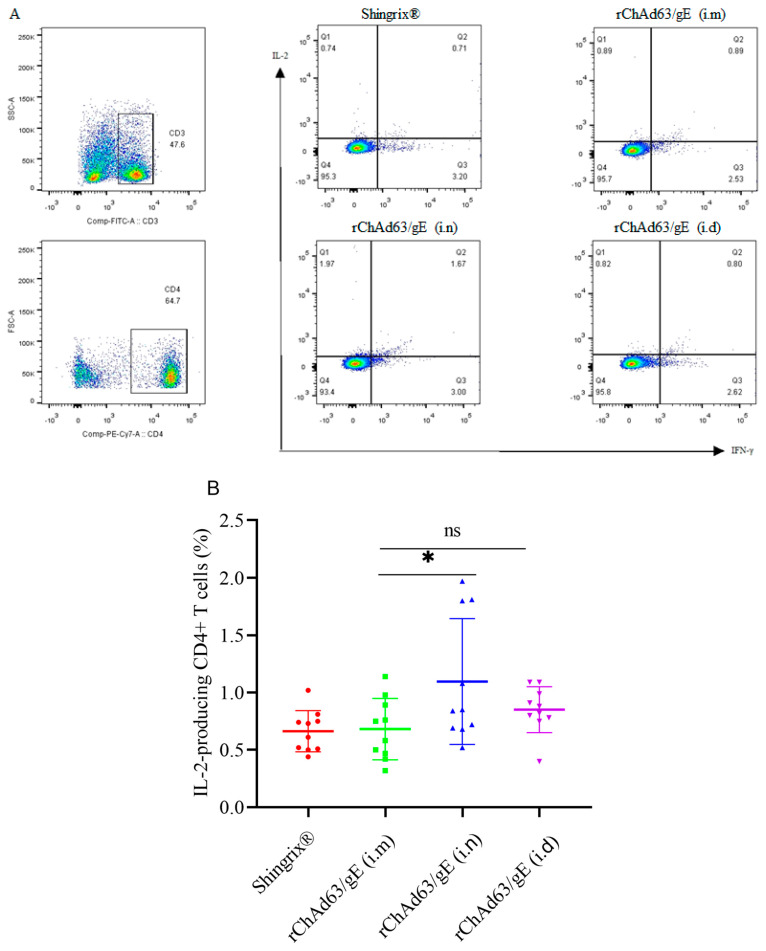

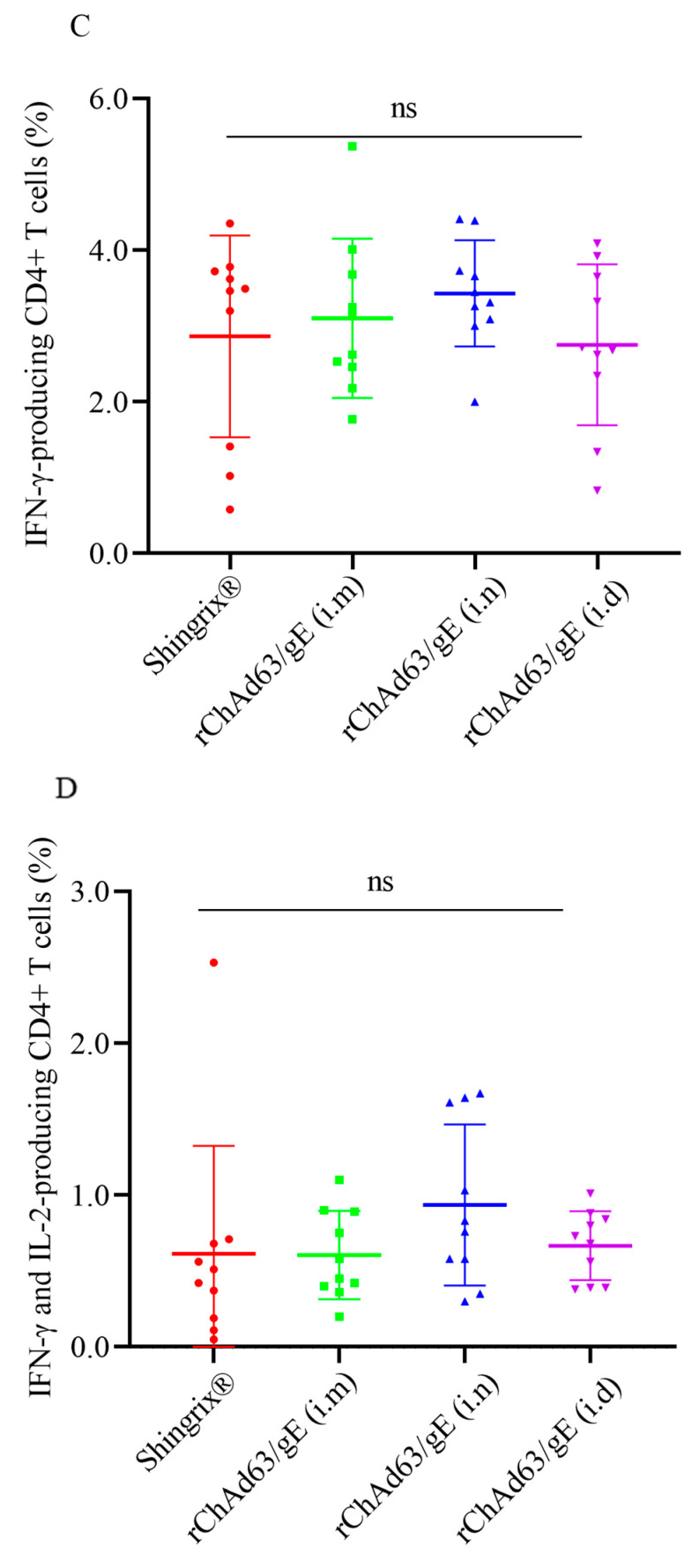
The functional CD4^+^ T cell immune response in C57BL/6 mice induced by recombinant adenoviruses. C57BL/6 mice (n = 10) were immunized with recombinant adenovirus. On the 14th day after booster immunization, splenocytes were isolated and stimulated with pooled peptides, and the number of IFN-γ and/or IL−-2-secreting CD4^+^ T cells was detected using flow cytometry. (**A**) Representative flow cytometric dot plots of CD4^+^ T cells for expressing IFN-γ and/or IL−2, (**B**) the number of IL−2-secreting CD4^+^ T cells, (**C**) the number of IFN-γ-secreting CD4^+^ T cells, (**D**) the number of IFN-γ- and IL−2-secreting bifunctional CD4^+^ T cells. Data were one of two independent experiments and shown as mean ± SD, *: *p* < 0.05, ns: no statistical difference.

In order to comprehensively analyze the induced CMI, especially the function of CD4^+^ T cells after vaccination, flow cytometry was further used to detect the cytokine expression of CD4^+^ T cells stimulated in vitro using pooled peptides (Figure 4). The CD3, CD4, IL-2 and IFN-γ antibodies were labeled with FITC, PE/Cyanine7, APC and PE, respectively. The lymphocytes were first gated on the basis of FSC and SSC, and the T cells were then gated on the basis of the surface expression of CD3. Thirdly, CD4^+^ T cells were gated on the basis of the PE/Cyanine7 fluorescent label, respectively. Finally, the secretion of IFN-γ or IL-2 by T cells were gated on the basis of the PE or APC fluorescent label, respectively. IL-2-secreting CD4^+^ T cells were in the Q1 region, IFN-γ and IL-2-secreting bifunctional CD4^+^ T cells were in the Q2 region and IFN-γ-secreting CD4^+^ T cells were in the Q3 region (Figure 4A). Compared with the i.m injection group, both the i.n and i.d injection groups induced similar numbers of IFN-γ-secreting CD4^+^ T cells (*p* > 0.05). Compared with the i.m injection group, the i.d injection group induced a similar number of IL-2-secreting CD4^+^ T cells (*p* > 0.05), but the i.n group induced more IL-2-secreting CD4^+^ T cells (*p* < 0.05).

Multifunctional T cells can express multiple cytokines at the same time, which are associated with protective immunity against many viral diseases and play an important role in recovery after viral infection. Therefore, we further analyzed the number of IFN-γ and IL-2-secreting bifunctional CD4^+^ T cells, and the results (Figure 4D) showed that, compared with the i.m injection group, both the i.n and i.d injection groups induced similar numbers of IFN-γ and IL-2-secreting bifunctional CD4^+^ T cells (*p* > 0.05).

### 3.4. Efficient Induction of gE-Specific IFN-γ and/or IL-2-Secreting CD8^+^ T Cells in Mice Immunized with Recombinant Adenoviruses

**Figure 5:**
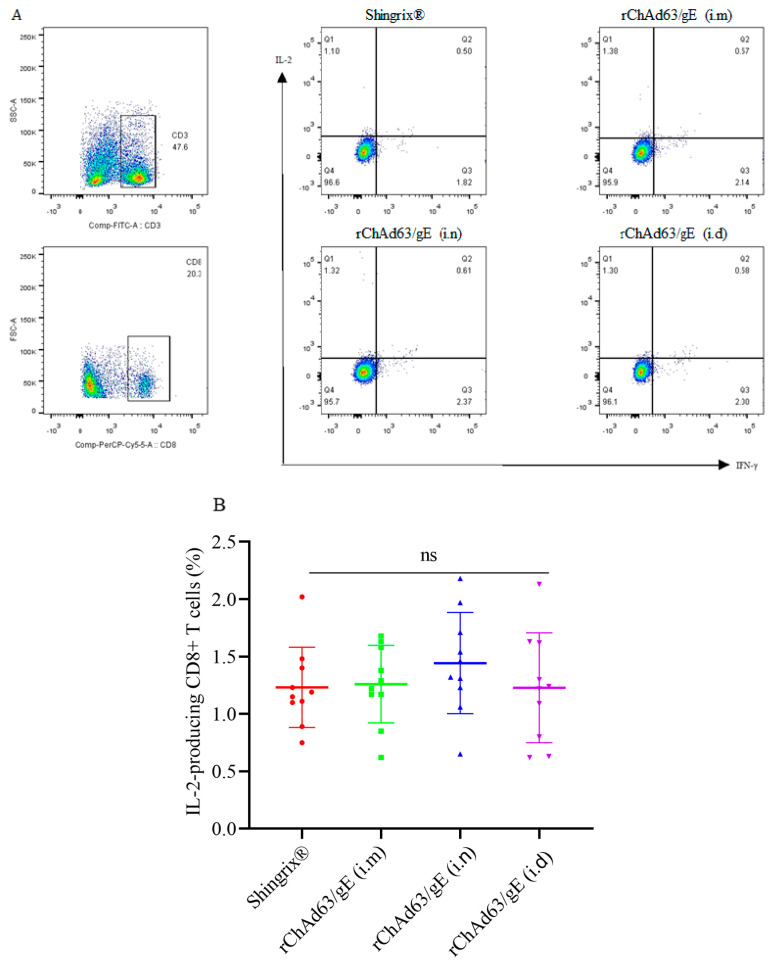

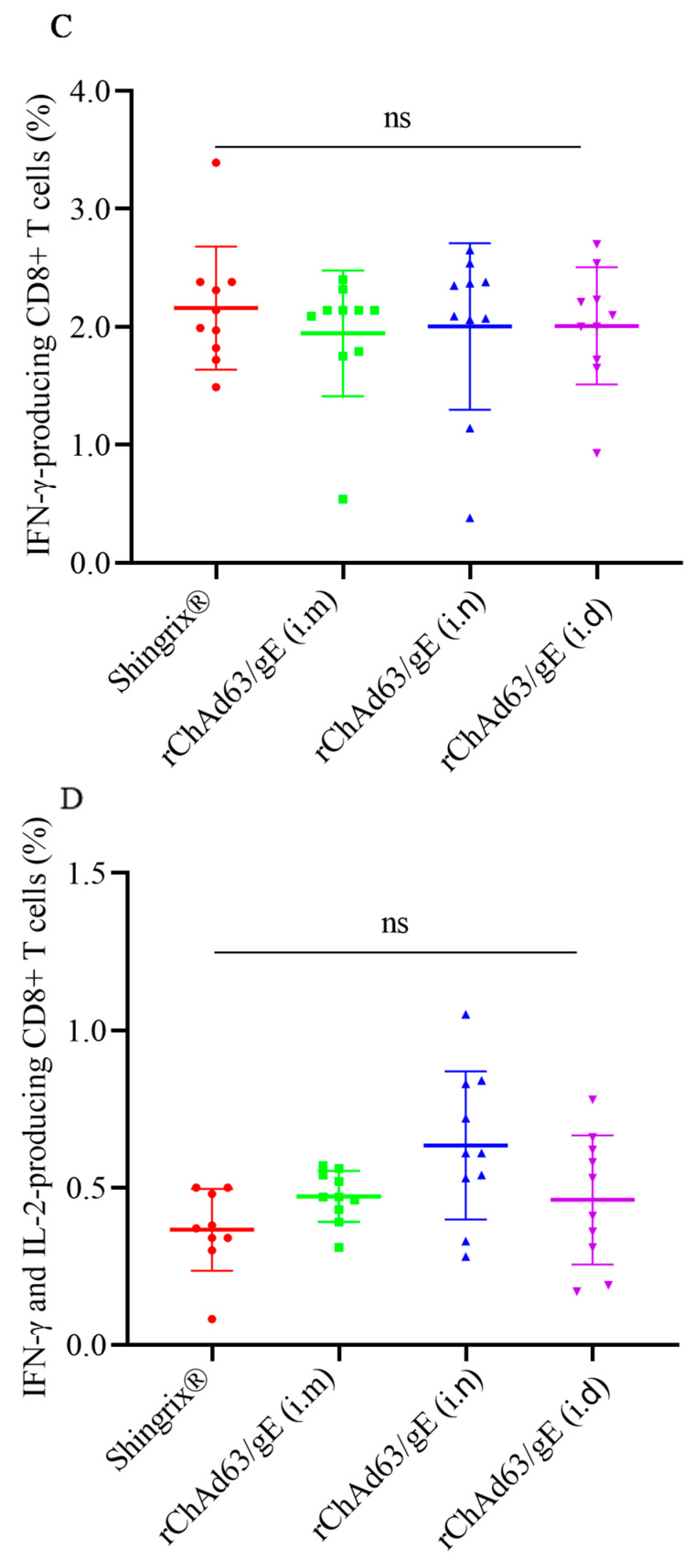
The functional CD8^+^ T cell immune response in C57BL/6 mice (n = 10) induced by recombinant adenoviruses. (**A**) Representative flow cytometric dot plots of CD8^+^ T cells for expressing IFN-γ and/or IL−2, (**B**) The number of IL−2-secreting CD8^+^ T cells, (**C**) The number of IFN-γ-secreting CD8^+^ T cells, (**D**) The number of IFN-γ and IL−2-secreting bifunctional CD8^+^ T cells. Data were one of two independent experiments and shown as mean ± SD, ns: no statistical difference.

In this study, flow cytometry was further used to detect the cytokine expression of CD8^+^ T cells stimulated in vitro using pooled peptides (Figure 4). The lymphocytes were first gated on the basis of FSC and SSC, and the T cells were then gated on the basis of the surface expression of CD3. Thirdly, the CD8^+^ T cells were gated on the basis of the PerCP/Cyanine5.5 fluorescent label, respectively. Finally, the secretion of IFN-γ or IL-2 by T cells was gated on the basis of the PE or APC fluorescent label, respectively. The results showed that similar numbers of gE-specific IL-2-producing CD8^+^ T cells, gE-specific IFN-γ-producing CD8^+^ T cells or IFN-γ and IL-2-producing CD8^+^ T cells were detected in each group (*p* > 0.05) (Figure 5).

## 4. Discussion

Here, we first reported the immunogenicity of recombinant adenovirus vectors of rChAd63/gE expressing gE protein by vaccinating C57BL/6 mice, and investigated the humoral and cellular immune responses instigated by homologous adenovirus-vectored vaccines via different immune pathways. The level of gE-specific functional CD4^+^ T cells and CD8^+^ T cells in mice following immunization with homologous rChAd63/gE did not display any difference from Shingrix^®^. So, recombinant adenoviruses expressing VZV gE are likely an alternative choice that warrants being further developed as a vaccine candidate against the onset of HZ. Similar CD4^+^ T and CD8^+^ T cell responses were induced in mice vaccinated with rChAd63/gE via different immune pathways, which indicated that i.n and i.d injection were also suitable immune routes for a herpes zoster vaccine based on adenovirus.

There were two vaccines, Zostavax^®^ and Shingrix^®^, previously licensed for the prevention of HZ, but Zostavax^®^ was stopped in the United States on 1 July 2020. Currently, only Shingrix^®^ is available for the prevention of HZ. Shingrix^®^ could elicit a highly immunogenic responses and durable protection. However, it also results in 8.5–17.0% vaccine recipients reporting a grade 3 severe reaction at the immunization site, as well as systemically [14,16]. Additionally, Shingrix^®^ could also cause Guillain–Barré syndrome [40]. Therefore, it is of great importance for the development of vaccines to prevent HZ that have a lower reactogenicity or reduced adverse events.

Adenovirus vectors have been widely used as vaccine vectors because of their advantages, such as a wide range of host cells, good safety, high replication titer, promotion of secretion of inflammatory cytokines, differentiation of immature dendritic cells and no need for adjuvants. During this pandemic of SARS-CoV-2, an adenovirus-vectored vaccine such as Ad26. COV2-S displayed good vaccine efficacy (VE) against COVID-19 disease and was successful in generating an effective spike-specific CD4^+^ T cell response following single-dose immunization [34]. Although adenovirus-based SARS-CoV-2 vaccines being administered intramuscularly can lead to the rare serious side effect of vaccine-induced thrombotic thrombocytopenia (VITT), it has been suggested this kind of adverse event may be avoided by preventing accidents in intravenous injection or using an intranasal route when administering such vaccines [41]. Furthermore, until now, no cases of the side effects of adenovirus-based SARS-CoV-2 vaccines have been found among vaccine recipients from developing countries. In this study, we investigated whether mice immunized with rChAd63/gE could induce the desired humoral and cellular immune responses. The results showed that similar CD4^+^ T and CD8^+^ T cell responses to those observed with Shingrix^®^ were induced in mice vaccinated with rChAd63/gE via different immune pathways, which elucidates that recombinant adenoviruses expressing VZV gE could be appropriate for further development as a new HZ vaccine candidate. The in vivo experiment results also showed that rChAd63/gE i.n or i.d immunized C57BL/6n mice could induce CD4^+^ T and CD8^+^ T cell immune responses similar to that for i.m injection. The results indicated that the use of a recombinant adenovirus expressing VZV gE protein via i.n or i.d injection were two promising strategies for HZ vaccine immunization.

For HZ vaccines, CMI plays a crucial role in inhibiting latent VZV activation, so the key to HZ vaccines is long-lasting and effective CMI [19]. Furthermore, it has been shown that the VZV-specific CD4^+^ T cell response is not only important in limiting the onset of HZ but also frequently adopted as a good indicator for the potential of zoster vaccines [42,43,44]. In this study, rChAd63/gE induced a similar detectably bifunctional CD4^+^ T cell response compared to Shingrix^®^. Previous studies have shown that the IL-2-secreting CD4^+^ T cells induced by the HZ vaccine are central memory T cells (TCM), while the IFN-γ and IL-2-secreting bifunctional CD4^+^ T cells are effector memory T cells (TEM) [45]. The IFN-γ from the CD4^+^ T cells is an essential cytokine to control VZV infection [46,47], but the only IFN-γ-secreting CD4^+^ T cells are terminally differential, are short-lived cells, have limited proliferative capacity and cannot provide long-term protection [47,48,49]. The IL-2 from the IL-2-secreting CD4^+^ T cells can promote the expansion of T cells and endow T cells with a memory function. Although we still need further evidence to support the induced durable protective effect, the appearance of IFN-γ- and IL-2-secreting bifunctional CD4^+^ T cells is beneficial to maintain long-term effective immune protection. Finally, it is worth emphasizing that although the values of gE-specific T cells capable of secreting cytokines analyzed here using flow cytometry are not high, similar data were observed in a previous investigation based on the same animal model carried out by GSK, and the percentage of gE-specific T cells secreting cytokines was around 1–2% after stimulation even with a pool of peptides spanning the gE antigen [39]. Moreover, it is consistent with the reports about the low VZV-specific CD4^+^ responses from a zoster vaccine [25,30,50].

In addition, in this study, homologous prime-boost vaccination strategies using rChAd63/gE could induce both CD4^+^ T cell and CD8^+^ T cell immune responses in C57BL/6 mice, suggesting the recombinant adenovirus vector expressing gE protein also possessed an ability to induce a CD8^+^ T cell response. This is not surprising when considering the adenoviral vector vaccine encoding the gE gene can de novo synthesize gE protein in the infected host cells, result in intracellular presentation by MHC I molecules as well as trigger CD8^+^ T cell responses. Recently, an HZ mRNA vaccine and a PLGA nanoparticle vaccine also induced gE-specific CD4^+^ T and CD8^+^ T cell responses, which is consistent with the results of this study [12,44].

There were also some shortcomings in this paper. Although the underling mechanism of protection has already been proven to be the cell-mediated immunity rather than neutralizing antibodies, the neutralizing antibodies should be further detected. Additionally, the duration of the response should be analyzed. Generally, to evaluate the induced protection, we can challenge the vaccinated mice with VZV infection. However, this is infeasible because VZV cannot efficiently replicate in animal models, which does not support assessing the efficacy of a gE candidate vaccine against VZV pathogenesis, latency and reactivation in a preclinical setting [39].

In summary, we demonstrated that the recombinant adenovirus vectors of rChAd63/gE expressing gE protein were highly immunogenic in regard to generating gE-specific CD4^+^ and CD8^+^ T cell responses in C57BL/6 mice, and i.n, i.m or i.d immunization with rChAd63/gE induced similar humoral and cellular immune responses. These observations suggest that the recombinant adenoviruses expressing VZV gE could be promising vaccine candidates and a vaccination strategy against HZ, and i.n and i.d injection were also suitable immune routes for a herpes zoster vaccine based on adenovirus.

## Data Availability

The data presented in this study are available on request from the corresponding author.
